# Revisiting Radiographic L5-S1 Parallelism Using MRI T1 Mapping

**DOI:** 10.5334/jbsr.1501

**Published:** 2018-09-27

**Authors:** Julien Galley, Federico Balagué

**Affiliations:** 1 HFR Fribourg, CH

**Keywords:** Magnetic resonance imaging, Musculoskeletal, Spine, Intervertebral disc, T1 mapping

## Abstract

**Purpose:**

Thirty years ago, we reported that parallel aspect of the L5-S1 disc on a lateral view of the spine might be considered to be an initial stage of disk degeneration. The current study represents an attempt to increase the validity of parallel sign on conventional radiograph using MR real T1 mapping.

**Methods:**

Forty-four young asymptomatic volunteers (mean age 21.6 ± 2.3) underwent lumbar spine MRI, twice the same day, morning and afternoon. Dedicated sequences using the inversion-recovery technique were used to calculate the T1 relaxation time. A region of interest (ROI) representing the nucleus pulposus was defined in each disk. The volunteers were stratified according to the presence or absence of a parallel morphology of L5-S1. Correlation between endplates angles, sacral slopes and T1 values were then evaluated.

**Results:**

L5-S1 space looks parallel for angles <10° (mean value 6.9° ± 1.4°). Sacral slope was lower in parallel disks (31.7 ± 4.9° vs. 40.1 ± 5.6°), showing a significant difference of 8.4° (p < 0.05). The T1 relaxation values show a significant difference between the two groups (p < 0.05) with a difference of 96 ms for the morning (1090.9 ± 33.3 ms for the parallel group and 1186.9 ± 41.2 ms for the non-parallel) and 121.9 ms for the afternoon (respectively 1004.7 ± 22.2 ms and 1126.6 ± 12.9 ms).

**Conclusion:**

The difference between the two groups suggests that parallel morphology of the L5-S1 disk is associated with lower water content.

## Introduction

When imaging is required in cases of low back pain, the first-line imaging technique is still controversial [1]. While magnetic resonance imaging (MRI) is now widely and increasingly used, the distinction between incidental findings and current symptom etiology is sometimes difficult [2,3]. Nevertheless, MRI is undoubtedly considered the best available technique for the study of the intervertebral disc. However, MRI facilities are not equally available worldwide and particularly in some areas, patients have access to standard radiographs at best [4,5,6,7,8]. This conventional approach still plays a role in the evaluation of the bony structures of the lumbar spine and can show us some indirect signs about disc degeneration. These features are associated with low back pain [9,10].

In 1981, our group described a parallel aspect of the lumbosacral adjacent endplates as being associated with initial stages of L5-S1 disk disorders [11]. Very quickly, two Italian groups verified our findings and reached similar conclusions [12,13]. To our knowledge, all the studies focused on this specific phenotype are cross-sectional, which prevents us from drawing any conclusion about causal relationships between a parallel morphology and disc degeneration.

Different imaging techniques like T1ρ or T2 mapping [14,15,16] have been used to evaluate the biochemical modifications of the discs. T1 mapping has also been proven to be sensitive to water content [17,18,19,20]. Becoming increasingly familiar with the T1 mapping technique [20], we aimed at performing a validation trial to determine whether parallel morphology of the L5-S1 intervertebral disc is associated with biochemical differences compared with discs exhibiting the normal morphology, i.e., higher anteriorly than posteriorly on a lateral view.

## Material and Methods

### Participants

Recruitment for the study was from medical staff or acquaintances and university students.

Inclusion criteria for volunteers were: good health, absence of any back symptom and age between 18 to 25 years. Exclusion criteria were: medical history of back pain, radiculopathy or neurological deficit, back trauma, previous back surgery or infiltration, osteoarticular or connective tissue disease, body mass index of >25, contraindication to MRI.

All participants were asked to have normal daytime activity and to avoid any heavy work (not to bear weight over 10 kg) and any sport during the day of examination.

Written informed consent was obtained from all participants.

### Imaging

The methodology details have been reported elsewhere [20]. The examinations were performed between December 2014 and July 2015. All the volunteers were scanned twice the same day (once in the morning at 8 a.m. and once in the late afternoon around 5 p.m.) in a relaxed supine position. MR imaging was performed using a 1.5T MR unit (Optima 360 Advance, GE Healthcare, Waukesha, WI, USA). The standard MR protocol using sagittal T1-weighted fast spin echo and sagittal T2-weighted fast spin echo sequences was performed. Dedicated sequences were then realized for T1 relaxation time measurements, using the inversion recovery technique with different inversion recovery times (from 100 to 2500 ms).

### Measurements

#### Parallelism

**Figure 1:**
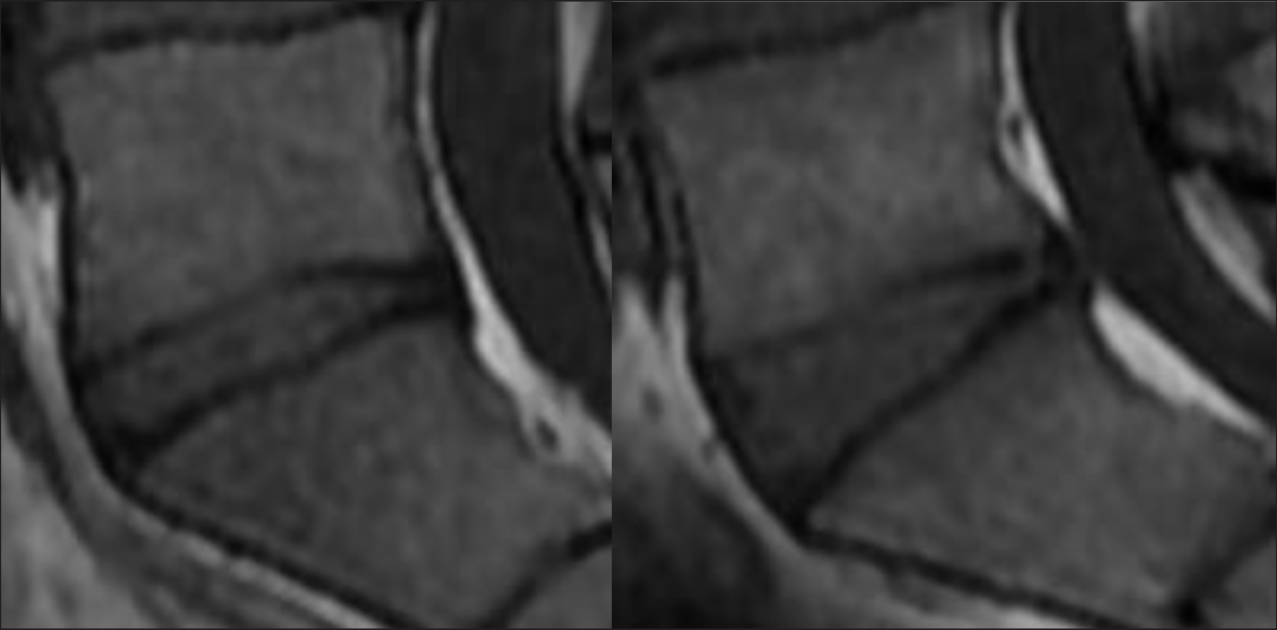
Examples of parallel and non-parallel L5-S1 disks.

The L5-S1 discs were evaluated on MRI sagittal T1 slices. On the sagittal medial plane, the angle made by the two endplates (Figure 1) were measured in each of the 44 volunteers. We then evaluated observer ability to classify the disc morphology as parallel or non-parallel at a glance, without measurement. As considered in the previous study, a disc space would appear parallel if the angle made by the two endplates is less than 10°. The inter-observer agreement was assessed with 14 discs. Seven discs measured less than 10° (mean 6.9 ± 1.4) and seven discs more than 10° (mean 16.4 ± 3.3). The images were anonymized and randomly evaluated. The two observers then had to classify them as parallel or non-parallel on the sagittal median slice and para-median (left and right, for a total of 42 images). The observers examined the images twice on the same day to evaluate intra-observer reliability.

#### Sacral slope

**Figure 2:**
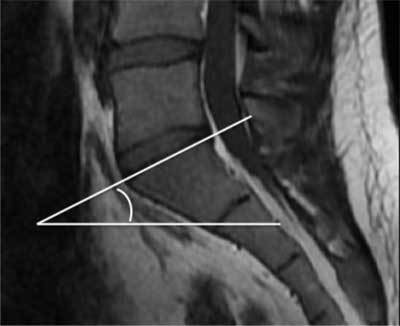
Example of sacral slope measurement.

Was defined by the angle made by the line along the superior of endplate of S1 and the horizontal line [21] (Figure 2).

#### T1 Mapping

**Figure 3:**
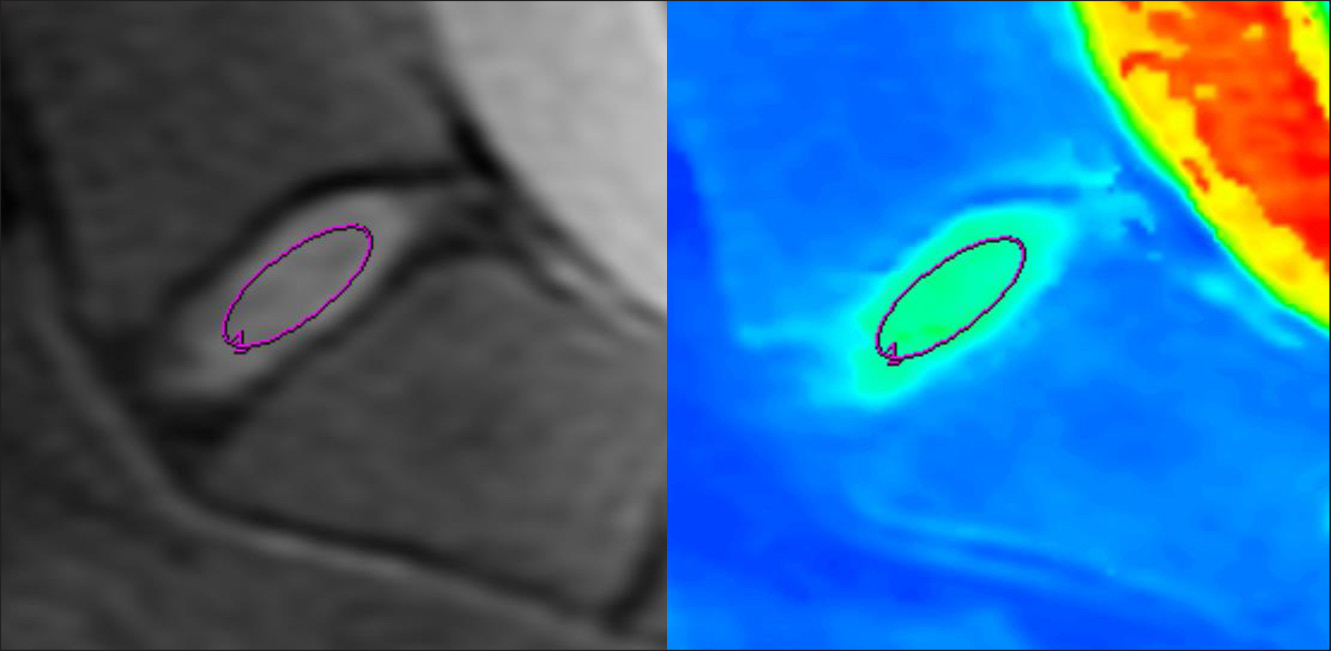
Example ROI placement representing the nucleus pulposus area and the corresponding mapping image.

As in the previous study, we defined a region of interest (ROI) representing the nucleus pulposus. Two virtual horizontal lines of the outer border of each endplate were defined. An ovoid ROI between those lines (range 45–75 mm^2^), less than half of the length of the disc, centered on the middle, was considered to be the nucleus pulposus area (Figure 3).

### Ethical committee approval

This study was approved by the CT CER (Communauté de travail des Commissions Suisses d’éthique pour la recherche sur l’être humain, Lausanne), which is the regional ethical committee for our hospital.

## Results

Forty-four healthy and asymptomatic volunteers were included in this study: mean age 21.6 ± 2.3, age range 18–25 years, 21 females and 23 males.

All the discs were classified according to the Pfirrmann classification. Forty-one were classified grade I or II and three discs grade III. Grades I and II discs were considered representative of non-degenerative discs and used for measurements.

The different measured values are displayed in Table 1. For the parallel group (n = 7), the sacral slope mean value was 31.7 ± 4.9°, the L5-S1 angle 6.9 ± 1.4°, the T1 values 1090.9 ± 33.3 ms for the morning and 1004.7 ± 22.2 ms for the evening. Respectively, the values for the non parallel group (n = 34) were: 40.1 ± 5.6°, 14.3 ± 2.1°, 1186.9 ± 41.2 ms and 1126.6 ± 12.9 ms.

**Table 1:** Values (mean ± Standard Deviation) of the parallel (N = 7) and non-parallel groups (N = 34).

	Sacral slope (°)	L5-S1 angle (°)	T1 values (ms)	

			Morning	Evening

Parallel (n = 7)	31.7 ± 4.9	6.9 ± 1.4	1090.9 ± 33.3	1004.7 ± 22.2
Non parallel (n = 34)	40.1 ± 5.6	14.3 ± 2.1	1186.9 ± 41.2	1126.6 ± 12.9
P value	<0.05	<0.05	<0.05	<0.05

T-tests were performed to analyse the difference between the two groups. The sacral slope and the L5-S1 angle show a significant difference (p < 0.05) of respectively 8.4° and 7.4°. The T1 measured values show a significant difference (p < 0.05) between the two groups with lower values for the parallel groups in the morning (difference of 96 ms) as well as in the afternoon (difference of 121.9 ms).

For the evaluation of parallelism, the intra-observer reliability was >0.9 (40/42). The inter-observer reliability was also excellent at >0.9 (38/42, with agreement on the cases after discussion).

## Discussion

A parallel L5-S1 disc cannot be considered the usual phenotype. A recent study on a cohort similar to ours but of Asian origin has shown that the L5-S1 disc has the greatest segmental lordosis of all the lumbar discs [22]. This finding is in agreement with the usual radiographic morphology of the L5-S1 disc, which appears higher anteriorly than posteriorly rather than parallel in a lateral view of the spine [23,24].

Our results show a decreased T1 relaxation time in parallel discs compared with those with a “normal” non-parallel phenotype. Thus, we can postulate that lower L5-S1 angle (parallelism), which is associated with decreasing T1 value, suggests early sign of disc degeneration.

In asymptomatic individuals, the sacral slope is around 40° [25], and it has been shown that patients with disc herniation or degenerative disc problems exhibit an angle about 5° smaller [26]. With a difference of 8.4°, our findings are in agreement with these results.

Our study has some limitations that reduce the generalizability of the findings. First, our subjects were young (≤25 years) and declared themselves asymptomatic. Consequently, the data might not necessarily be the same in a cohort of patients. The same caveat could apply to elderly individual, as it has also been reported that diffusion patterns in lumbar discs of asymptomatic subjects are significantly age-related [27]. The link between images and clinical variables is another challenge not addressed in this study [28]. However, Fenty et al. [29] reported that T1ρ values of the nucleus pulposus as well as disc height are significantly decreased in painful discs.

Moreover, the parallelism was initially described using standing lateral radiographs of the spine. For reasons of radiation exposure, standard radiographs were not ordered in this study. Before starting the analysis, we performed a preliminary comparison of the L5-S1 angle in some subjects (n = 28) that had undergone standard radiographs and an MRI less than 24 hours apart and found minor differences between the two techniques in this regard. In addition, it has been shown that MRI measurements of lumbar disc height and volume have sufficient validity and reliability [30].

## Conclusion

A parallel phenotype of the L5-S1 disc likely should be considered abnormal in the sense of an initial stage of dehydration and possibly degeneration. Investigating the implications of this radiographic phenotype in a clinical context should be encouraged. This might be of relevance for areas of the world where access to MRI is limited.
